# Methods for Conjugating Antibodies with Quantum Dots

**DOI:** 10.3390/molecules30193999

**Published:** 2025-10-06

**Authors:** Pavel Sokolov, Alexander Knysh, Irina Kriukova, Pavel Samokhvalov, Yury V. Kistenev

**Affiliations:** 1 Laboratory of Optical Quantum Sensors, Life Improvement by Future Technologies (LIFT) Center, Skolkovo, 143025 Moscow, Russia; a.knysh@lift.center (A.K.); i.krukova@lift.center (I.K.); p.samokhvalov@lift.center (P.S.); 2 Laboratory of Nano-Bioengineering, National Research Nuclear University MEPhI (Moscow Engineering Physics Institute), 115409 Moscow, Russia; 3 Department of Clinical Immunology and Allergology, Institute of Molecular Medicine, Sechenov First Moscow State Medical University (Sechenov University), 119146 Moscow, Russia; 4 Laboratory of Laser Molecular Imaging and Machine Learning, National Research Tomsk State University, 36 Lenin Av., 634050 Tomsk, Russia; yuk@iao.ru

**Keywords:** antibody, quantum dot, conjugate, site-specific, site-nonspecific

## Abstract

Nanomaterials are increasingly used in the development of detection systems for various disease biomarkers as tools for reliable early diagnosis, which is a key factor in reducing mortality and increasing treatment effectiveness. The use of quantum dot–antibody conjugates allows for optical detection of various disease markers in biological fluids, tissues, and individual cells with high sensitivity and specificity. The sensitivity and specificity of detection are determined not only by the outstanding optical properties of fluorescent quantum dots but also by the type of antibodies used for binding target analytes and the methods of their conjugation with quantum dots. This review deals with methods of site-specific and site-nonspecific conjugation of quantum dots with antibodies, including full-length and single-domain antibodies, as well as antibody fragments, with a special focus on their structural features and active moieties used for binding to their targets. The review includes examples of successful applications of quantum dot–conjugated antibodies in diagnosis, environment monitoring, and food safety assessment. We also discuss the prospects of further research in this field, including new conjugation methods and issues related to the stability and specificity of probes. The review provides a comprehensive analysis of the current methods and achievements in antibody conjugation from the viewpoint of subsequent analyte detection, highlighting the importance of further research for improving the existing technologies.

## 1. Introduction

Recent studies show that expanding the scope of screening and the introduction of new, more sensitive diagnostic methods can reduce mortality, delay disease progression, mitigate disease severity, and prolong recurrence-free survival in oncological [1], neurodegenerative [2], infectious [3,4], and other diseases. A traditional diagnostic method employs a biological capture molecule, which binds the analyte and a reporter label, which is detected by instrumental methods. Various antibodies (Abs), peptides and nucleotide aptamers [5,6,7], molecularly imprinted polymers [8], and other compounds [9] are used to detect protein markers of diseases, Abs being the most common. They are typically conjugated with various optical labels, such as fluorescent organic dyes [10] or quantum dots (QDs) [11], enzymatic labels (e.g., in ELISA) [12], radioactive labels, as well as labels for in vivo detection by positron emission tomography, computed tomography [13], or magnetic resonance imaging [14]. Fluorescent detection is one of the most sensitive detection methods, QDs being the best fluorescent labels due to their unique optical and physicochemical properties [15].

The history of methods for conjugating Abs with fluorescent labels based on organic dyes dates back to studies by Albert Coons et al. in the 1940s [16]. Over the past decades, many techniques have been developed, and the conjugation of Abs with organic fluorescent labels has become a routine task, although the conjugation procedures still impair the affinity of the Abs and reduce the quantum yield of the dyes [17]. The first conjugates of QDs with Abs were obtained as recently as the late 1990s [18]; since then, the number of papers describing the conjugation methods has been growing exponentially. There are examples of the use of QD–Ab conjugates in fluorescent immunohistochemical analysis [19], immunoassay [20], flow cytometry [21], solid microarrays [22,23], and various biosensors [24,25] for the detection of pathogenic biological objects [26], as well as in in vitro and in vivo imaging of cells [27], organs, and tumors [28]. However, the obtaining of active QD–Ab conjugates still has not become a routine procedure because of problems with the selection of QD ligands serving as conjugation linkers, as well as issues related to the stability of the conjugates and the preservation of Ab activity [29].

**Figure 1:**
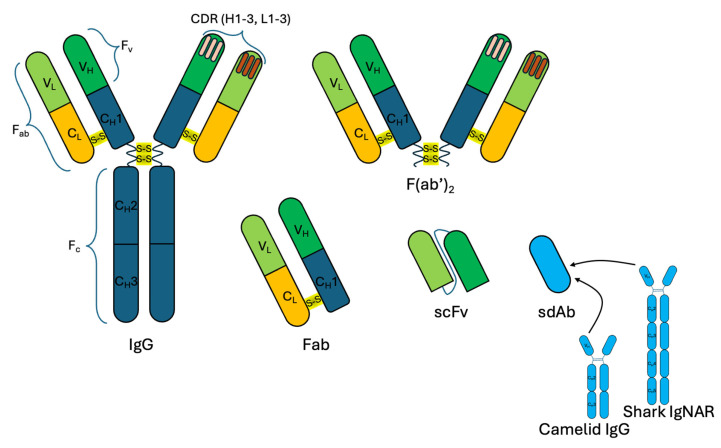
Antibodies and their structural elements used as biological capture molecules. IgG, immunoglobulin G; Fab, fragment antigen-binding region; F(ab′)_2_, two Fab regions connected by disulfide bonds in the hinge region of the antibody; scFv, single-chain variable fragment; sdAb, single-domain antibody; IgNAR, new antigen receptor antibody; F_c_, crystallizable fragment; F_v_, variable fragment; CDR, complementarity determining region; V_L_, light chain variable domain; C_L_, light chain constant domain; V_H_, heavy chain variable domain; C_H_, heavy chain constant domain.

Full-length class G immunoglobulins (IgG) are most commonly used as biological capture molecules. They are obtained by immunizing laboratory animals, such as rabbits, mice, and rats, to subsequently isolate and purify a pool of polyclonal Abs (pAbs). If monoclonal Abs (mAbs) are required, B cells are collected from immunized animals and then somatically fused with myeloma cells using various techniques [30]. The Abs produced by the resulting hybridomas are then screened, and those with the highest specificity and affinity for the immunization antigen are selected [31]. Unlike pAbs, which are a set of antibodies that bind to different epitopes of the same antigen, mAbs obtained from a single hybridoma culture bind to a single specific antigen epitope, which ensures reproducible results of analysis and easy scaling of production. Both mAbs, which have reproducible properties in detecting target analytes, and pAbs, which recognize a number of epitopes within a single antigen, thus increasing the detection sensitivity, are used for detection [32]. Fragments of Abs, including Fab and F(ab′)_2_ fragments obtained by site-specific cleavage of full-length Abs [33], are also used (Figure 1). In addition to hybridoma-derived Abs, recombinant Abs are increasingly often used as new biotechnological methods are being developed. They are obtained by expressing the nucleotide sequences encoding the Abs in bacterial strains and mammalian cell lines. Both classical full-length IgG and their fragments, including scFV [34], bispecific Abs [35], trispecific Abs [36], etc., as well as single-domain Abs (sdAbs) representing the variable domain of the heavy chain of camelid Ig (VHH) [37], or fragments of the variable domain of shark Ig (shark Ig new antigen receptor (IgNAR) Abs) are obtained using this approach [38].

**Figure 2:**
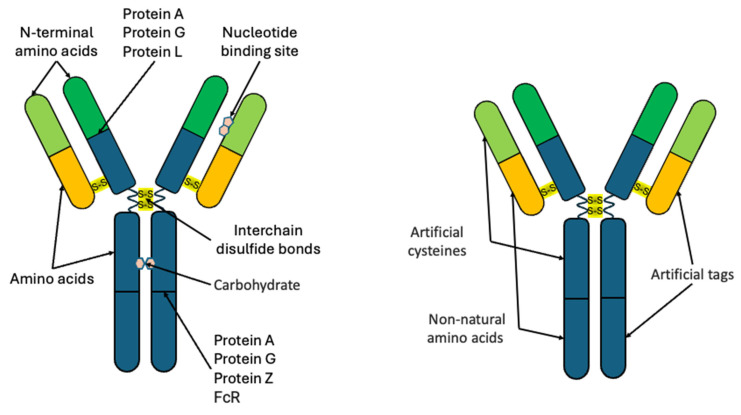
Site-specific and site-nonspecific binding of antibodies to reporter molecules. FcR, the receptor that binds the antibody Fc region.

Recombinant antibodies are expressed in cell lines, such as CHO [39] and HEK293 [40], as well as yeasts [41] and bacteria [42]. Expression in yeasts and bacteria reduces the cost and shortens the time of obtaining recombinant Abs [43], although it may not ensure correct folding and the normal post-translational modifications, or even may entail abnormal post-translational modifications [44]. At the same time, the production of Abs by recombinant methods allows the introduction of special active groups and tags into them in order to ensure their site-specific conjugation with reporter labels (Figure 2). Most existing detection systems employ full-length Abs or their fragments due to their availability, although there is a growing number of developments of recombinant Abs and examples of their use for the detection of target analytes.

The optical and physicochemical properties of QDs and organic fluorescent dyes differ substantially. Most fluorescent dyes are excited only in a limited wavelength range (with an absorption peak width at half height typically in the range of 30–100 nm), whereas QDs have a continuous absorption spectrum with a maximum in the ultraviolet region, as well as a large molar absorption coefficient and a two-photon absorption coefficient that is several orders of magnitude higher than that of organic fluorophores. Therefore, they can be excited in a wide wavelength range, which makes it possible, e.g., to reduce background excitation of biological tissues and fluids and, hence, increase the signal-to-noise ratio. The symmetrical and narrow fluorescence spectra of QDs, as well as the large Stokes shift, allow them to be used for simultaneous multiplexed detection of several analytes. The order of magnitude greater photostability and fluorescence lifetime of QDs compared with organic fluorescent dyes allows the accumulation of the QD fluorescence signal and its time-resolved detection, which also increases the signal-to-noise ratio and, accordingly, the detection sensitivity. At the same time, although QDs are one to two orders of magnitude larger than organic fluorescent dye molecules, QDs are equally suitable for labeling intracellular analytes [45,46]. The only possible disadvantage of using QDs as reporter labels for detection is that they are somewhat more difficult to conjugate with biological capture molecules.

There are reviews on the conjugation of antibodies with QDs, such as the paper by Foubert et al. [47], which describes the methods available at that time for the conjugation of antibodies and QDs, as well as the reviews by Yemets et al. [48] and Kang et al., where strategies of the chemical conjugation of proteins are analyzed in detail [49]. However, most reviews focus on the application of antibody–QD conjugates to cancer theranostics [50] and diagnostics and to monitoring the effectiveness of therapies for various diseases [51,52]. Here, we consider published examples of obtaining QD–Ab conjugates, examine the ligands used for QD functionalization and approaches to site-specific and site-nonspecific conjugation, and present our vision of the further development of QD–Ab conjugation techniques. About 30% of the articles, described in this review, has been published in last three years, which give the information about modern conjugation approaches. About 30% of the articles on modern conjugation approaches cited in this review have been published in the last three years.

## 2. Site-Nonspecific Conjugation of Antibodies with Quantum Dots

### 2.1. Noncovalent Binding Methods

**Figure 3:**
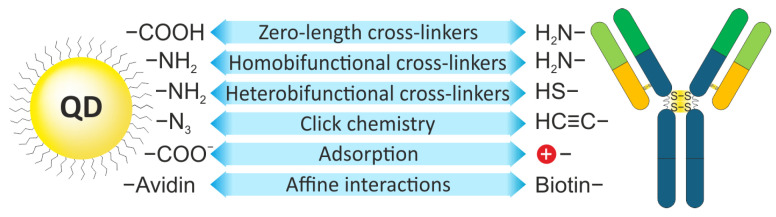
Methods of site-nonspecific binding of antibodies to reporter molecules.

Site-nonspecific conjugation of QDs with Abs is the most common type of conjugation, because it requires minor modifications to the QD surface and Ab structure. This approach can be implemented in one of the following ways (Figure 3). The simplest but not the most effective type of QD–Ab conjugation is physical absorption driven by electrostatic, as well as hydrophilic or hydrophobic, interactions between the Abs and the organic ligand shell of the QDs. Accordingly, the efficiency of conjugation depends on the pH and ionic strength of the solution, surface charge of the ligand shell, and even the temperature. To transfer QDs into the aqueous phase, hydrophilic ligands are used, whose free groups also determine the surface charge of the QDs. The density of coating of the QD surface with the ligands determines not only the hydrophilic/hydrophobic properties of the QDs, but also the efficiency of protein adsorption on their surface. At the same time, despite the simplicity of conjugation by physical absorption, Abs conjugated by this method often lose their activity due to their partial denaturation as a result of interaction with the nanoparticle shell, as shown by Torcello-Gomez et al. [53], and the conjugates themselves may be unstable when buffers, pH, ionic strength, or temperature are changed.

There is an approach providing highly reliable and stable QD–Ab conjugation without using covalent immobilization. This approach is based on the interaction of affinity pairs, such as streptavidin–biotin. This method is very popular, and researchers often use commercially available QDs functionalized with avidin/streptavidin for conjugation with biotinylated Abs [54,55]. In addition, there are many variations in this method, allowing, e.g., control of the amount of avidin/streptavidin on the QD surface. For example, Goldman et al. [56] used avidin-functionalized CdSe/ZnS QDs for conjugation with biotinylated Abs. They synthesized QDs whose surface was coated with dihydrolipoic acid (DHLA) to impart hydrophilicity and a negative charge to their surface. To functionalize the QD surface, the authors used a mixture of avidin and a translational fusion of maltose binding protein and the leucine zipper interaction domain, which allowed them to control the amount of avidin on the QD surface. The surface was functionalized through electrostatic interaction between the positively charged avidin, positively charged leucine zipper interaction domain, and negatively charged DHLA. The Abs were biotinylated by site-nonspecific labeling of Abs with a long-chain derivative of biotin N-hydroxysuccinimidyl ester. As a result, the authors obtained conjugates of Abs against cholera toxin with QDs emitting at 520 nm and conjugates of Abs against staphylococcal enterotoxin B with QDs emitting at 590 nm, whose activity was confirmed in a multiplexed ELISA.

In addition to electrostatic functionalization of the QD surface with avidin, which does not provide permanent immobilization, there are approaches based on covalent functionalization of the QD surface with streptavidin. Chen et al. [57] compared classical ELISA and QD-based fluorescence ELISA. They used CdTe QDs functionalized with 3-mercaptopropionic acid (MPA) to make them hydrophilic. Covalent functionalization of the QD surface with streptavidin was performed using carbodiimide chemistry, with a QD-to-streptavidin molar ratio of 1:2. The resulting conjugates were purified from unreacted components by ultrafiltration. Covalent site-nonspecific biotinylation of Abs was performed at lysine amino acid residues using the NHS–LC–biotin linker and was followed by purification via centrifugation in a concentrator. The resulting conjugates were used in ELISA. They ensured a detection limit of 3.8 ng/mL, which increased the detection sensitivity by a factor of 5.5 compared with conventional ELISA. Pathak et al. [58] compared the numbers of Abs per QD in the conjugates obtained using the streptavidin–biotin pair and using covalent conjugation of Ab fragments to QDs via the succinimidyl 4-(N-maleimidomethyl)cyclohexane-1-carboxylate (SMCC) linker. Biotinylated Abs contained an average of four to eight biotin molecules attached at random sites throughout the Ab, which, presumably, resulted in conjugation of IgG molecules with QDs in all possible spatial orientations. The authors found that an average of 0.60 ± 0.14 or 1.3 ± 0.35 Ab molecules was adsorbed to one QD in the case of streptavidin–biotin conjugation at an initial Ab molecule to QD ratio of 1:1 or 2:1, respectively, in the mixture. In the case of direct covalent conjugation, each QD adsorbed as few as 0.076 ± 0.014 Ab molecules at both initial ratios in the QD–Ab mixture. This is substantially lower than the estimates previously reported in the literature [59].

### 2.2. Covalent Binding Methods

The second type of site-nonspecific conjugation is covalent immobilization. Unlike physical adsorption and affinity interaction methods, this approach ensures permanent immobilization. Covalent immobilization employs QDs whose surface is functionalized with organic ligands that have carboxyl (–COOH) or amino (–NH_2_) terminal groups.

**Figure 4:**
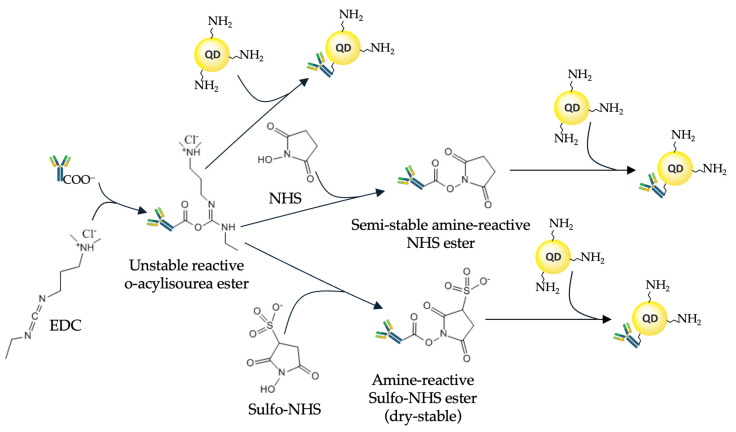
Schematics of covalent conjugation of antibodies with quantum dots using carbodiimide chemistry.

Most often, ethylene dichloride (EDC) is used for this purpose. It activates the carboxyl groups of the QD ligand shell, allowing them to bind to primary amines of the Ab molecule. Two main conjugation strategies employ EDC: direct activation of the carboxyl group and activation aided by N-hydroxysulfosuccinimide (NHS) or sulfo-NHS. EDC reacts with a carboxyl group to form an active intermediate compound, O-acylisourea, which is easily displaced by means of nucleophilic attack of a primary amino group in the reaction mixture, the primary amine forming an amide bond with the original carboxyl group (Figure 4). The EDC byproduct is released in the form of a soluble urea derivative. NHS, or sulfo-NHS, is used to increase the efficiency of conjugation and stabilize the amino-reactive intermediate (O-acylurea). The addition of sulfo-NHS converts O-acylisourea into an amino-reactive sulfo-NHS ester, thereby increasing the efficiency of the conjugation reaction. However, primary amines of both the N-terminal amino acid and the side chains of the about 80 lysine residues throughout the IgG Ab molecule [60,61] can be involved in the conjugation. All these primary amines are positively charged at physiological pH values and are predominantly located on the surface of the Ab molecule, which makes them accessible for conjugation. This leads to chaotic orientation of the Abs upon conjugation and may block their antigen-binding sites. A detailed protocol for the conjugation of QDs functionalized with ligands that have free carboxyl groups and Abs, via free amino groups of the Ab amino acid residues, has been developed by East et al. [62].

**Figure 5:**
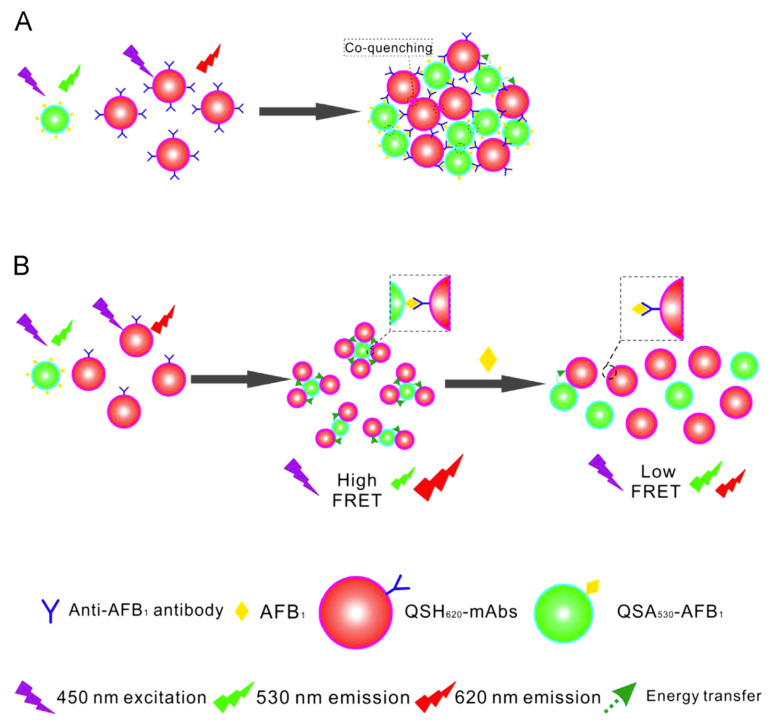
Schematic representation of the competitive homogenous QDs-FRET immunosensing strategy for the determination of AFB_1_. Multi-antibodies labeled QSH_620_ may result in an un-controllable FRET-based immunoassay (**A**) due to excess Abs binding sites lead to chain aggregation between respective QDs via the antigen–antibody interaction. (**B**) The well-designed competitive immunoassay for AFB_1_ based on FRET between multi-AFB_1_ conjugated QSA_530_ and monovalent-Ab labeled QSH_620_. This figure is not drawn to scale. Reproduced with permission from Xu et al. [67]. Copyright © 2014 Elsevier B.V.

Liang et al. have developed a QD-based fluorescence-linked immunosorbent assay (FLISA) for high-throughput detection of apramycin (APR) [63]. They used commercial water-soluble carboxyl-functionalized ZnCdSe/ZnS QDs for EDC-mediated conjugation with amino groups of monoclonal antibodies against APR. In experiments with pure APR, the limit of detection (LoD) was 0.38 ng/mL. In samples of animal tissues, such as swine kidney and bovine muscle, contaminated with APR, the LoD was 2.3–5.7 μg/kg which is significantly better than that of the conventional ELISA (7.5–18.75 μg/kg). The QD–Ab conjugates are used in many FLISAs (for more examples, see the review by Lv et al. [64]). Wu et al. have developed a QD-based lateral flow assay (LFA) for the detection of ciliary neurotrophic factor (CNTF) [65]. They used commercially available CdSe/ZnS QDs functionalized with carboxylic groups for EDC-mediated conjugation with recombinant anti-CNTF antibodies. The LFA contained a conjugate pad impregnated with QD-labeled anti-human CNTF antibodies interacting with the analyte in a liquid sample. The capture antibody for human CNTF was fixed at specified sites of the membrane to capture the mixture of analyte and QD-labeled detection antibodies. The LoD of CNTF LFA was as low as 6.45 pg/mL, which is comparable with that of the traditional ELISA (6.42 pg/mL). More examples of using QD–Ab conjugates in LFA are presented in a recent review by Ahmad Najib et al. [66]. Due to the broad absorption and narrow emission spectra of QDs, the QD–Ab conjugates are often used in FRET-based biosensors. For example, Xu et al. have developed an immunosensor for detecting aflatoxin B_1_ (AFB_1_) based on FRET between QDs of different sizes [67]. They used QDs modified with a carboxylic acid that fluoresced at a wavelength of 620 nm (QSH_620_) for EDC-mediated coupling with anti-AFB_1_ antibody (with a conjugation efficiency of approximately 0.84 anti-AFB_1_ mAbs per QD) and QDs modified with amino groups that fluoresced at 530 nm (QSA_530_) for conjugation with AFB_1_. The operation principle of the immunosensor is shown on Figure 5. The immunosensor provided a LoD of 0.13 pM (0.04 ng/mL) for AFB_1_ in rice extracts, the overall immunosensor performance being comparable with that of the conventional ELISA. More examples of QD-based biosensors employing FRET are summarized by Grazon et al. [68] and Shamirian et al. [69].

The –NH_2_ and –COOH groups of the QD surface shell ligands are charged, which can lead to nonspecific binding of QDs to cells and biological molecules. Therefore, researchers suggest various alternatives to QD surface ligands that have a neutral charge. For example, Han et al. [70] have developed ligands with electrically neutral free groups for this purpose. Polyimidazole ligands incorporating norbornene (NBPILs), based on a copolymer of imidazole and polyethylene glycol (PEG), effectively coated the QD surface, maintaining the quantum yield of QD fluorescence at 80–90% in aqueous solutions. Tetracycline-modified Abs were obtained by reacting tetracycline derivatives with lysine residues in the Ab molecule, after which they were immediately mixed with the NBPIL–QD solution. This yielded conjugates with a hydrodynamic diameter of about 15–17 nm, which were purified from unconjugated Abs by gradient centrifugation. The conjugates exhibited a low level of nonspecific staining of peripheral blood mononuclear cells in flow cytometry, as well as intense specific staining in multiplexed cell staining experiments in vivo.

**Figure 6:**
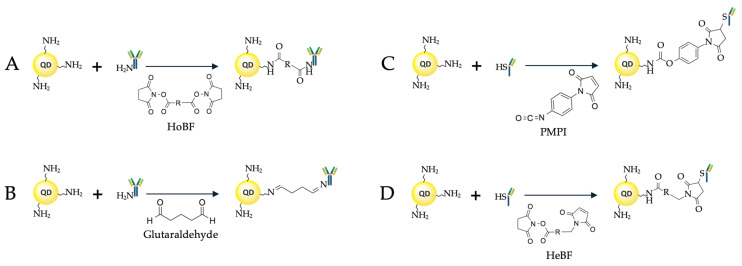
Covalent conjugation of antibodies with quantum dots (**A**) using a homobifunctional coupling agent (HoBF) containing an NHS ester at each end; (**B**) using glutaraldehyde; (**C**) using a heterobifunctional coupling agent (HeBF) containing amine-reactive and sulfhydryl-reactive groups; (**D**) using alcohol–thiol coupling.

Bis(sulfosuccinimidyl)suberate (BS3) is another example of a short linker used for covalent conjugation of Abs to the QD surface [71] (Figure 6A). For conjugation, the authors used commercially available QDs (Qdot™ 605 ITK™, Thermo Fisher Scientific, Eugene, OR, USA) functionalized with PEG derivatives with a terminal amino group (NH_2_-QDs), which were mixed with BS3 at a molar ratio of 1:1000, the excess unreacted crosslinker being removed by centrifugation with molecular filters after 30 min of incubation. After that, anti-CD44 Abs were added to the QD solution in PBS at ratios of 1:3, 1:5, 1:10, and 1:12, and the reaction mixture was incubated for another 2 h. At the final step, a glycine solution was added to inactivate unreacted BS3 molecules on the QD surface, and the resulting conjugates were purified by centrifugation with 300 kDa molecular filters. To test the activity of the resulting conjugates, the surface plasmon resonance (SPR) method was used, with CD44 immobilized on the SPR sensor disk. It was found that a maximum of about four Ab molecules could be adsorbed on one QD at the QD-to-Ab ratio of 1:10, which is a high conjugation efficiency.

When short linkers are used for QD–Ab conjugation, there is a risk of disrupting the Ab structure due to nonspecific interactions (electrostatic and hydrophobic/hydrophilic ones) with the QD surface. However, this also makes it possible to obtain ultrasmall conjugates, which more readily penetrate cells and, hence, can detect intracellular analytes. In this case, it is possible to use adapter molecules that are initially conjugated to the QD surface, and then Abs are attached to them. This approach is described by Sahoo et al. [72], who used QDs functionalized with bovine serum albumin (BSA). BSA was conjugated via carboxyl groups of the QD ligand shell, which were activated by means of carbodiimide chemistry using carbonyldiimidazole (CDI). CDI is a highly reactive carbonylating agent containing two acylimidazole groups. This crosslinker reacts with carboxylate to form an active N-acylimidazole group capable of binding to molecules containing amines, forming a stable amide covalent bond (Figure 6B). Functionalization of QDs with BSA allows other biological molecules to be attached by activating aldehyde groups. The Abs were then oxidized with periodate and mixed with the activated QDs for conjugation by forming covalent bonds. The BSA-modified QDs were stable for at least 12 weeks; the resulting conjugates were 20.8 ± 5.4 nm in size as estimated by transmission electron microscopy. The functional activity of the conjugates was studied by staining cell lines in vitro and by means of ELISA; the dependence of the antigen detection efficiency on the number of QD-conjugated Abs was also estimated. A similar approach to the conjugation of Abs with CdTe QDs covalently functionalized with BSA at carboxyl groups of the QD ligand shell was described by Oliveira et al. [73]. To synthesize the conjugates, the authors also initially used sodium periodate as an oxidizing agent for the alcohol groups of N-glycans of Abs against CA19-9, which were thus converted into reactive aldehydes capable of binding to the amino groups of BSA. The authors used the obtained conjugates to engineer a biosensor that allowed quantitative detection of CA19-9 with a wide dynamic range and high sensitivity.

The procedure of Ab conjugation through streptavidin–biotin interaction can also be adapted for site-specific binding of Abs. For example, there are approaches to site-specific biotinylation of Abs using biotinylated photoactivated labels in combination with the immunoglobulin-binding domain of protein A [74,75], which will be discussed in more detail in the next section.

In addition, an original approach to spatially nonoriented conjugation preserving the activity of antigen-binding sites was proposed by Dvorakova et al. [76]. First, the authors prepared magnetic particles with molecules of the target analyte, apolipoprotein E (ApoE), covalently immobilized on their surface. Then, pAbs against ApoE were added to the functionalized magnetic particles. When the immune complex formed and the unbound Abs were washed off, the mixture was activated with EDC and sulfo-NHS, and conjugation with carboxylated QDs was performed. The conjugates were purified by magnetic separation and destruction of the antigen–Ab immune complex by adding 0.05% trifluoroacetic acid and 0.5% SDS. This procedure ensured that Abs conjugated with QDs retain their antigen-binding properties.

## 3. Site-Specific Conjugation of Antibodies with Quantum Dots

**Figure 7:**
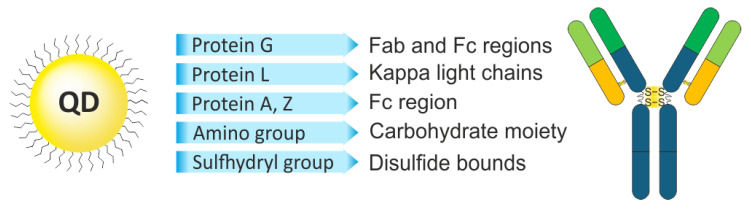
Methods of site-specific binding of antibodies to reporter molecules.

One of the main tasks in obtaining QD–Ab conjugates is to preserve the antigen-binding properties of Abs, for which purpose various site-specific conjugation approaches are used. These approaches, discussed in detail below, typically require additional adapter molecules, including various affinity proteins, to restrict conjugation to specific sites in the Ab amino acid sequence (Figure 7).

### 3.1. The Use of Affinity Proteins

Various bacterial proteins that interact with Ab sites and serve for protecting against the host’s immune system can be used for site-specific binding. For example, protein A binds to the Fc domain of the Ab, interacting with IgG heavy chains. Jin et al. [77] used protein A as an adapter for site-specific binding of Abs to QDs. CdSe/CdZnS QDs were coated with glutathione (GSH) as a ligand, and covalent binding to protein A was carried out using EDC/sulfo-NHS. The QD to protein A ratio was 10; unreacted sulfo-NHS groups were inactivated by the addition of PEG-NH_2_, so that the QD surface was coated with protein A and PEG-NH_2_. Because the size of QDs affects their biodistribution and pharmacokinetics, the authors used homemade QDs with hydrodynamic diameters of 4.4 and 6.8 nm (for QD–GSH), smaller than most commercially available QDs [78]. The hydrodynamic diameters of QDs functionalized with protein A were, respectively, 8.6 and 9 nm, and when Abs were added at a ratio of six Abs per QD, the diameter became about 20 nm. The functionality of the QD–Ab conjugates obtained by this method was demonstrated in experiments on multiplexed staining of HER2 and CXCR4 in KPL-4 cells. The hydrodynamic diameter of the conjugates can be reduced by using the IgG-binding Z domain of protein A instead of the complete protein. The molecular weight of the Z domain is only 6.5 kDa, less than 20% of the molecular weight of protein A, and the IgG-binding affinity of Z domain–based dimers is an order of magnitude higher than that of full-length protein A [79,80]. Makride et al. [81] obtained a Z domain dimer with a hexahistidine tag for purification by means of metal affinity chromatography and a tag for in vivo biotinylation, which was then used to functionalize streptavidin-coated CdSe/ZnS QDs. The QDs functionalized with the Z domain dimer were then mixed with Abs for conjugation, and the functional activity of the conjugates was verified by Western blot staining. The advantages of this approach are the ease of conjugation with Abs without the need for individual labeling of different Abs and a higher affinity of the Z domain for the Fab domain compared with the full-length A protein [82].

In addition to protein A, which selectively binds Ig molecules, other Ig-binding proteins are used, such as proteins G and L. Native protein G also contains a domain that binds serum albumin; therefore, fragments of protein G that lack this activity are typically used to increase the binding capacity of Abs [83]. Tsuboi et al. [84] used the Ig-binding domain B1 of protein G (GB1) as an adapter for site-specific binding of Abs to QDs. The surfaces of CdSe/ZnS and CdSeTe/CdS QDs (QD600 and QD830, respectively) were functionalized with GSH to make them hydrophilic. GB1 molecules with a molecular weight as small as 9.3 kDa were produced in *Escherichia coli* as a translational fusion with the hexahistidine tag (HisGB1). The complex of QD and HisGB1 was formed through the interaction of the His tag with Zn^2+^ or Cd^2+^ ions on the surface of the QD shell, with an average of about 18–19 HisGB1 molecules per QD. This made it possible to obtain functionalized HisGB1-QDs with a hydrodynamic diameter of 8–9 nm and 10–11 nm for QD600 and QD830, respectively, and conjugates with Abs against HER2 with a size of 40–60 nm as estimated by dynamic light scattering. The QD–Ab conjugates obtained by this method were successfully used for detecting HER2 in KPL-4 cell culture by fluorescence microscopy and for detecting xenograft breast tumors in mice by injecting the conjugate solution into the tail vein. A similar approach was used by Goldman et al. [85], who also conjugated QDs with the Ig-binding domain β2 of protein G (PG) as an adapter. They used PG protein obtained in *E. coli* in the form of a translational fusion with a positively charged leucine zipper (PG-zb), which strongly interacted with the surface of CdSe/ZnS QDs coated with negatively charged dihydrolypoic acid (DHLA). The authors functionalized the QD surface with PG-zb and a translational fusion of maltose-binding protein and leucine zipper (MBP-zb) at different ratios. This allowed them to purify the conjugates on immobilized maltose, as well as to control the number of Ab conjugation sites and, hence, the number of Ab molecules per QD, by varying the MBP-zb to PG-zb ratio. For example, there were an average of 5.5 Abs per QD at an MBP-zb to PG-zb ratio of 1:2.4 and only 2.7 Abs per QD at a ratio of 1:0.8. The activity of the resulting conjugates was tested by ELISA using enterotoxin B (SEB) and 2,4,6-trinitrotoluene (TNT) as reference analytes.

Protein L, one of the envelope proteins of *Peptostreptococcus magnus*, efficiently binds not only with IgG, but also with IgM, IgE, and IgA via their light chains; however, it is only capable of binding with κ-type chains [86]. Lao et al. [87] engineered a three-component linker based on protein L for QD–Ab conjugation. The linker, expressed in *E. coli* in the form of a translational fusion, contained three components: a hexahistidine (His) tag for interaction with the QD surface through coordination interaction with Zn^2+^ ions of the QD shell, an elastin-like polypeptide representing 78 repeated VPGVG amino acid sequences for purification procedures, and protein L for binding IgG Ab against carcinoembryonic antigen (CEA). The VPGVG polypeptide can undergo a reversible phase transition from the soluble form to aggregates, e.g., upon temperature change, which is used for purification of proteins conjugated with it from aggregates [88]. This allowed the authors to skip the stage of chromatographic or affinity purification. The activity of the obtained conjugates was confirmed by CEA detection in Western blot and by immobilization of CEA on the surface of microarrays.

However, conjugation using protein A, G, or L is not covalent; hence, the conjugates may be disrupted by changes in the characteristics of the medium. Zhang et al. [89] proposed a versatile method for covalent immobilization using protein A. For this purpose, they used a linker consisting of a fragment of protein A with a p-benzoyl-L-phenylalanine (Bpa) photoactivatable group and an additional cysteine residue introduced in its Z domain. When the Z domain of protein A bound to the Ab, and Bpa was activated by ultraviolet radiation, covalent crosslinking occurred. The cysteine residue was used for covalent crosslinking with the CdSe/ZnS QD surface functionalized with amino and carboxyl groups by carbodiimide chemistry methods. The activity of the oriented conjugates of these QDs with Abs against shrimp allergen in immunochromatographic tests was six times higher than that of conjugates obtained by site-nonspecific binding.

### 3.2. The Use of Carbohydrates

Human IgG is glycosylated at the asparagine residue 297 (N297) of the heavy chain of the Fc domain. Ab glycans typically contain vicinal diol fragments that can be oxidatively cleaved to form aldehydes. Thus, conservative glycans can serve for site-specific conjugation. Wilkins et al. [90] compared site-specific conjugation via Fc-domain oligosaccharides and site-nonspecific conjugation using carbodiimide chemistry. The glycosylated region of the Ab was oxidized to obtain a binding site by adding sodium periodate, after which sodium sulfite was added to stop the reaction. The oxidized Abs were purified by ultrafiltration. For site-specific conjugation, the authors used amino-functionalized CdS/ZnS QDs, which were mixed with oxidized Abs at a molar ratio of 3:1 and incubated overnight. Sodium cyanoborohydride was then added to the reaction mixture in a 75-fold molar excess over Abs. After 30 min of incubation, ethanolamine was added to block unreacted sites. The resulting conjugates were purified by ultrafiltration. Site-specific and site-nonspecific conjugates were compared in tests on the detection of the N-terminal pro-B-type natriuretic peptide (NT-proBNP) using an immunochromatographic assay (ICA) developed by the authors. The results showed that conjugates obtained by site-specific binding of Abs provide an almost two times stronger signal ensuring a detection limit of 10 pg/mL, versus 80 pg/mL in the case of site-nonspecific conjugates obtained by carbodiimide chemistry methods.

### 3.3. The Use of Disulfide Bonds

The side chain of cysteine contains a thiol group (–SH), which can be covalent bound to the QD surface, usually using maleimide chemistry methods. 3-Maleimide propionic acid hydroxysuccinimide (NHS) ester, a bifunctional crosslinking agent, is often used for this purpose. It contains an NHS group for the reaction with amines and a maleimide group for the reaction with thiol groups. Owing to the NHS group, the linker can be attached to almost any primary or secondary amino group on the QD surface. The maleimide fragment facilitates the attachment to proteins and peptides via the cysteine –SH group, as well as to other thiolated molecules. IgG Abs typically contain 32 cysteine residues, which form 16 disulfide bonds, which determine the three-dimensional structure of the Abs and are responsible for their stability. For this reason, cysteine residues are typically used for the conjugation of Ab fragments. In Ab conjugation using maleimide chemistry, the key step is the formation of a covalent bond between the maleimide group and the thiol group of the cysteine residue of the Ab. This is a Michael reaction, where the nucleophilic thiol group attaches to the electrophilic double bond of maleimide, forming a stable thioether bond. Examples of the use of this approach for QD–Ab conjugation were reported by Bilan et al. [91] and Brazhnik et al. [92]. For conjugation, CdSe/ZnS QDs were used, whose surface was functionalized with PEG-based ligands with a terminal amino group. Disulfide bonds in full-length goat IgG against mouse Ig were reduced using 2-mercaptoethanolamine-HCl (2-MEA) or dithiothreitol (DTT), after which the functional Ab fragments were purified by affinity chromatography against mouse Ig. The Ab fragments were then conjugated with QDs activated with succinimidyl 4-[N-maleimidomethyl]cyclohexane-1-carboxylate (SMCC), the conjugation reaction being stopped by adding 2-mercaptoethanol (2-ME). The activity of the conjugates purified by gel filtration chromatography was confirmed by flow cytometry and dot blotting. In addition to SMCC, N-(p-maleimidophenyl)isocyanate (PMPI) can also be used as a crosslinking agent, which allows the conjugation of cysteine residues with QDs whose surface contains free hydroxyl groups [93].

Zhang et al. [94] compared the characteristics of site-specific conjugates obtained by Ab cleavage and two site-nonspecific conjugates obtained by carbodiimide chemistry methods. They used CdSe/ZnSe/ZnS QDs primarily functionalized with GSH (QD–GSH) for solubilization, as well as those additionally functionalized with carboxyl-PEG-carboxyl (QD–PEG). Site-nonspecific conjugation was performed with Abs against C-reactive protein (CRP) and epidermal growth factor receptor (EGFR). In site-specific conjugation, the succinimidyl valerate–PEG–maleimide (SMPEG) linker was used, and Ab fragments were obtained by treating full-size Abs with 20 mM DTT. The site-nonspecific QD–GSH–Ab conjugates exhibited a high rate of nonspecific binding in experiments on the detection of CRP by immunofiltration, whereas the site-nonspecific QD–PEG–Ab conjugates exhibited a significantly lower nonspecific binding, their fluorescence signal increasing proportionally to the CRP concentration. Thus, the PEG-based ligand significantly reduced the nonspecific binding of the conjugates. The use of PEG with a molecular weight of approximately 2 kDa increased the water solubility of QDs, reduced nonspecific binding, and improved the binding of Abs to targets by reducing steric hindrance. Moreover, site-specific conjugates had a CRP detection limit of 0.39 mg/L versus a limit of more than 1.5 mg/L for site-nonspecific conjugates. In EGFR detection experiments on A549 cells, site-nonspecific QD–PEG conjugates also exhibited a high nonspecific binding, whereas site-specific conjugates emitted minor nonspecific signals and strong specific ones. Note that this advantage of site-specific conjugates was observed despite the considerably larger average Ab density on the QD surface in the site-nonspecific conjugates than in site-specific ones (2.13 and 0.76 Abs per QD, respectively).

Apart from the cysteine residues that participate in the formation of disulfide bonds to stabilize the structure of Abs, some Abs also contain free cysteine residues, which can be used for conjugation with QDs [95]. In addition to native cysteine residues, additional cysteine residues can be introduced into the Ab structure using various biotechnological methods and used for conjugation of Abs with the QD surface. For example, it is possible to introduce point mutations into the nucleotide sequences encoding Abs in order to replace lysine residues with cysteine ones, as shown by Hwang et al. [96]. The introduction of a cysteine residue is also used for the conjugation of sdAbs with QDs. In this case, the additional cysteine is attached to the C-terminus of the sdAb peptide chain. This approach was demonstrated by Brazhnik et al. [93].

### 3.4. Conjugation of Antibody Fragments

Anderson et al. [97] suggested a method for conjugating sdAbs with CdSe/ZnS QDs whose surface was functionalized with DHLA. For this purpose, they expressed sdAb genes as a translational fusion at the C-terminus with three repeating hexahistidine sequences separated by a short spacer (HHHHHH-AGSAGVEHHHHHH-AGSAGVE-HHHHHH). This tag allowed the sdAb to be immobilized on the QD surface through interaction with the zinc ions of the QD shell during incubation in 10 mM sodium borate buffer (pH 9) and incubation on ice for 1 h at an optimal sdAb-His to QD ratio of 30:1. The QD–sdAb-His conjugates obtained by this method were used both in immunofluorescence detection of ricin, where they ensured about the same detection limits as conjugates of the same anti-ricin sdAb labeled with a conventional fluorophore, and in SPR analysis, where they were used to amplify the signal. Moreover, QD–sdAb-His conjugates proved to be effective reporter elements in SPR sandwich assays, providing more sensitive detection with an approximately tenfold signal amplification compared with unconjugated sdAb and two- to fourfold amplification compared with conjugates containing full-length Abs. At the same time, streptavidin-modified QDs site-nonspecifically conjugated with biotin-labeled sdAbs provided four times smaller signal amplification in SPR assays compared with QD–sdAb-His conjugates, which showed that the spatially oriented conjugates were more efficient.

Sukhanova et al. [93,98] proposed a variant of site-specific covalent conjugation of sdAbs and QDs. For this purpose, they used CdSe/ZnS QDs whose surface was functionalized in two stages: the QDs were solubilized first with a DL-cysteine mixture and then with PEG with SH– and OH– (HS-C_11_-EG_6_) or SH– and NH_2_– (HS-C_11_-EG_6_-NH_2_) end groups. After that, the QDs were conjugated with sdAbs against CEA containing a cysteine residue at the C-terminus as follows: the intramolecular disulfide bonds of the sdAbs were reduced using tris(2-carboxyethyl) phosphine, and then conjugation was performed via the reactions with sulfosuccinimidyl 4-(N-maleimidomethyl)-cyclohexane-1-carboxylate (sulfo-SMCC) for QDs carrying OH- and NH_2_-containing ligands on their surface and with N-(p-maleimidophenyl) isocyanate (PMPI) for QDs with OH-containing ligands (Figure 6C). The resulting conjugates were purified by gel filtration chromatography. The conjugates obtained by this technique had an extremely small hydrodynamic diameter (about 12 nm) and were successfully tested in experiments with fluorescence immunohistochemical staining of tissue sections and flow cytometry. Although the site-specific conjugation of sdAbs with QDs is relatively simple, there are examples of their site-nonspecific conjugation with the QD surface via cysteine residues using carbodiimide chemistry [99,100]. Fragments of the variable domain of shark immunoglobulins (shark IgNAR Abs) can be conjugated site-specifically or randomly, as can sdAbs derived from camelids [101].

Pedroso et al. [102] described the use of QD conjugates with short-chain variable fragment (scFv) Abs for the detection of the urokinase-type plasminogen activator receptor (uPAR) protein, which is overexpressed in many cancers. The authors used CdSe/CdS QDs coated with a polymer shell based on poly(acrylic acid)-co-poly(n-octylacrylamide)-co-poly(2-aminoethylacrylamide) (PAOA) to increase the stability and solubility. Conjugation was performed using the SpyTag–SpyCatcher pair [103]. SpyTag is a short peptide consisting of 13 amino acid residues that spontaneously reacts with the small SpyCatcher protein (12.3 kDa) to form a stable isopeptide bond. The scFv fragment was obtained as a translational fusion of the variable domains of the heavy and light chains labeled with SpyTag. For effective functionalization of the surface of polymer-coated QDs with the SpyCatcher protein, a point amino acid substitution of Cys for Ser35 was performed. Conjugation was carried out through simple mixing and incubation of SpyCatcher-functionalized QDs and SpyTag–scFv overnight. This yielded compact conjugates with a hydrodynamic diameter of about 24 nm, which were effectively internalized by cells and stained intracellular uPAR, as shown by fluorescence confocal microscopy.

In addition, Xu et al. [104] presented an example of the use of a QD conjugate with scFv Abs against GPR78 for inhibiting the growth of cancer cells. For covalent conjugation, the authors used QD-625 activated with the heterobifunctional crosslinker 4-(maleimidomethyl)-1-cyclohexanecarboxylic acid N-hydroxysuccinimide ester (SMCC) and pre-reduced DTT scFv generated in *E. coli* (Figure 6D); the conjugates were purified by gel filtration. The functional activity of the conjugates was demonstrated by fluorescence immunohistochemistry and Western blot in cell line cultures and mouse xenograft tissues with implanted cancer cells.

Kolossov et al. [105] obtained compact conjugates of QDs and Fab fragments of Abs with a size of about 12 nm. They synthesized (Hg_x_Cd_1−x_Se) Cd_z_Zn_1−z_S QDs coated with a polymer shell based on polyacrylamide(histamine-co-triethylene glycol-co-azidotriethylene glycol) (P(IM-N3)) to increase the stability and biocompatibility. To obtain F(ab′)_2_ fragments, full-length IgG was cleaved with pepsin and then purified using protein A immobilized on agarose. F(ab′)_2_ was reduced to Fab using a 100 μM TCEP solution for 1 h at 37 °C, after which the resulting Fab fragments were purified by centrifugation in concentrators. The resulting Fab fragments were then functionalized with a tenfold molar excess of dibenzylcyclooctyne-PEG4-maleimide (DBCO-PEG4-maleimide), and full-length Abs used as a comparison system for conjugation with QDs were functionalized with DBCO-sulfo-NHS. The conjugates were obtained by mixing full-length Abs and Fab fragments in PBS with QDs and incubating at 4 °C overnight. Unreacted DBCO was neutralized by adding a 50-fold molar excess of 2-azidoacetic acid. To impart a negative charge to the conjugates, they were further treated with a mixture of taurine and (1R,8S,9s)-bicyclo[6.1.0]non-4-yn-9-ylmethyl N-succinimidyl carbonate (BMSC). For comparison, conjugates of full-length Abs and Fab fragments with the organic dye Alexa Fluor 647 (Thermo Fisher Scientific Inc., Waltham, MA, USA) were also obtained. The resulting conjugates of QDs with full-length Abs had a hydrodynamic diameter of about 22 nm, whereas the conjugates with Fab fragments were as small as 12.3 nm, only slightly larger than the QDs themselves (7.5 nm). Intracellular imaging of tubulin in HeLa cells using fluorescence microscopy and in mouse brain tissue samples using immunohistochemistry showed that spatially oriented conjugates of Fab fragments with QDs were the most efficient among the fluorescent probes tested.

Umakoshi et al. [106] studied the cleavage of Abs by tris(2-carboxyetheyl)phosphine hydrochloride (TCEP) and conjugation of the resultant fragments with maleimide-functionalized QDs at the single-molecule level using high-speed atomic force microscopy. The TCEP concentrations in these experiments ranged from 0.5 to 50 mM. The highest yield of half-molecule Ab fragments (approximately 75 kDa) was observed at a TCEP concentration of about 5 mM. At a TCEP concentration of 50 mM, most of the Ab fragments were heavy chains. The efficiency of conjugation with QDs also depended on the degree of Ab cleavage. In the case of partial Ab cleavage (TCEP concentration, 1 mM), 80% of QDs were not conjugated with Ab fragments, whereas at a TCEP concentration of 2 mM, most QDs were conjugated with at least one Ab fragment. The average number of Ab fragments conjugated with one QD increased with increasing TCEP concentration from 3.3 ± 1.4 at a TCEP concentration of 2 mM to 4.3 ± 1.5 at 5 mM. Thus, the ratio of Ab fragments available for conjugation and the absence of partly uncleaved Abs are important for conjugation with QDs.

Data on the Ab conjugation process are crucial for designing highly active conjugates. Site-specific conjugation considerably better preserves the affinity of antibodies and ensures the most efficient binding to the analyte. Maintaining antibody specificity and affinity is a key condition in obtaining antibody–drug conjugates (ADC). Note that many mechanisms of the conjugation of antibodies with drugs are the same as those employed in fabricating QD–Ab conjugates. Examples are kinetic model structures for the conjugation of a cysteine-modified mAb with a maleimide-functionalized surrogate drug described by Andris et al. [107] and the fabrication of cysteine-conjugated ADCs reported by Weggen et al. [108]. In addition, advanced approaches employing artificial intelligence are used to increase the effectiveness of antibody–drug conjugation. They are reviewed in a recent study by Noriega et al. [109].

## 4. Prospective Conjugation Technologies

This section describes technologies that are used to conjugate Abs with proteins, active pharmaceutical substances, organic fluorescent dyes and nucleic acids that have proven efficient and can be used for conjugation of Abs with QDs.

### 4.1. Nucleotide-Binding Sites

Nucleotide-binding sites (NBSs) are conserved regions of the variable domains of Abs (Fv). NBSs have affinity for nucleotides and some aromatic amino acids, which allows them to be used for site-specific conjugation. Mustafaoglu et al. [110] used the tryptamine-EG8-TA linker, which specifically binds to NBS, and lipoamido-dPEG8, which contains two sulfur atoms for interaction with the surface of gold nanoparticles. Covalent crosslinking between the linkers and Abs was performed through ultraviolet irradiation of a pre-incubated mixture of the Abs and linkers. This approach can also be adapted for site-specific conjugation of Abs to the QD surface by selecting the corresponding QD ligands containing tryptamine as a terminal group.

### 4.2. Aptamers

Aptamers are artificial DNA/RNA or amino acid sequences capable of specifically binding various molecules, which allows them to be used instead of Abs in biosensor applications [111]. However, aptamers are also suitable for site-specific conjugation of Abs. Soxpollard at al. [112] suggested that DNA aptamers be used for covalent immobilization with the Fc domain of mouse IgG1, IgG2a, and other IgG classes. They developed an NHS-modified DNA aptamer that specifically binds to the Fc domain of Abs, after which covalent crosslinking is performed by means of a carbodiimide reaction. This approach can be used, e.g., for site-specific labeling of Abs with biotin for subsequent conjugation with streptavidin-functionalized QDs.

### 4.3. Enzymatic Conjugation

Abs can be conjugated to QDs via glutamine residues using microbial transglutaminase (MTGase), an enzyme that catalyzes the formation of covalent bonds between the γ-carboxamide group (–CONH_2_) of glutamine residues and primary amines. This method allows site-specific labeling while preserving the antigen-binding activity of Abs. For example, in developing Ab–drug conjugates, transglutaminase-mediated modification of glutamine residues was used to obtain specific and stable conjugates. This approach ensures conjugation at specific sites, minimizing changes in the antigen-binding regions of Abs and preserving its specificity and affinity. Although there are no examples of using glutamine residues for QD–Ab conjugation, the principle of transglutaminase-mediated conjugation is well understood and can be applied to QD–Ab conjugation strategies. Jeger et al. [113] demonstrated that deglycosylated Abs could be site-specifically conjugated with substrates containing lysine or its derivatives, as well as with an NHS-modified fluorescent dyes, via glutamine residues at positions 295 and 297 in the Fc region.

### 4.4. Peptide Tags

MTGase can also be used to perform conjugation via artificial peptide tags introduced into Ab molecules. For example, Strop et al. [114] demonstrated the conjugation of fluorescent dyes with Abs via glutamine-containing short peptide tags (LLQG). They introduced the glutamine peptide tag into various regions of the crystallization domains of the heavy and light chains of Abs against EGFR and showed that the efficiency of site-specific conjugation with the fluorescent dye Alexa Fluor 488–cadaverine was as high as two dye molecules per Ab molecule.

Ramakrishnan et al. [115] developed an approach for site-specific conjugation of scFv fragments of Abs against HER2 by introducing a peptide containing 1, 3, or 17 threonine amino acid residues at the C-terminus. After the expression of scFv with a peptide tag, it was glycosylated with galactose (2-keto-Gal) using human polypeptide-α-Ν-acetylgalactosaminyltransferase II (h-ppGalNAc-T2) and conjugated with N-aminomethyl-carbonylhydrazino-Alexa 488. The functional activity of the conjugates obtained in this way was demonstrated using flow cytometry. Thus, this method can also be adapted for the conjugation of Abs with QDs whose surface is functionalized with ligands containing an amino oxide group.

An LPXTG peptide tag (X = D, E, A, N, Q, or K) can also be introduced into Abs for their conjugation with fluorescent dyes. This tag is recognized by enzymes of the sortase family that catalyze the cleavage of the polypeptide chain at the LXPTG site and the formation of a peptide bond between the resulting product and an exposed glycine residue of another protein. This approach has been successfully used for the conjugation of Abs with fluorescent labels [116] and can be adapted for glycine-functionalized QDs.

Another example of artificial tags used for site-specific QD–Ab conjugation is the SNAP tag. It is a genetically engineered mutant O6-alkylguanine transferase catalyzing the transfer of an alkyl group from a guanidine substrate to a cysteine residue in its active center. The SNAP tag (19.4 kDa) in the form of a translational fusion was attached to the C- or N-terminus of the protein to be conjugated, after which QDs functionalized with benzylguanine were added to the solution of the resulting hybrid protein [117,118]. This approach can also be used to conjugate QDs with Ab fragments.

### 4.5. Tyrosine Amino Acid Residues

Tyrosine residues contain a phenolic hydroxyl group, which can interact with isocyanates or carbodiimides. The molecules of Abs, in particular IgG, contain many tyrosine residues, especially in the variable regions. These residues are crucial for antigen recognition and binding. In complementarity-determining regions (CDRs), tyrosine accounts for approximately 10% of the total number of amino acid residues and is involved in about 25% of antigen contacts [119]. Therefore, when tyrosine residues are used for conjugation, the antigen-binding properties of Abs are likely to be impaired. However, the high abundance of tyrosine allows simultaneous site-specific labeling of a protein molecule with several fluorescent dye molecules [120]. In addition, there are examples of successful conjugation of Abs with various molecules via tyrosine residues, mainly those located in the Fc region of the Ab [121]. Methods for conjugating various protein molecules at tyrosine amino acid residues are reviewed in detail by Szijj et al. [122].

### 4.6. Non-Natural Amino Acids

The development of the technology for producing recombinant Abs has made it possible to obtain Abs with artificially introduced non-natural amino acids. These are also used to produce site-specific conjugates of Abs with drugs and fluorescent labels. To date, as many as 70 different non-natural amino acid residues has been reported to be introduced into protein molecules by cellular and extracellular expression methods. However, two non-natural amino acids are most commonly used for conjugation: p-acetylphenylalanine (pAcF) and p-azido-phenylalanine [123]. For example, Hutchins et al. [124] introduced p-acetylphenylalanine into the Fab fragment of the Ab against HER2 to perform site-specific conjugation with the fluorescent dye Alexa Fluor 488. For this purpose, 20 μM of the Fab fragment of the Ab against HER2 modified with the non-natural amino acid was incubated with 300 μM aminooxy-Alexa Fluor 488 and 100 mM methoxyaniline in acetate buffer (pH 4.5) at 37 °C. After 16 h of incubation, the unreacted dye was removed by centrifugation on molecular filters. This procedure ensured a conjugation efficiency as high as 81%.

## 5. Conclusions and Prospects

Although there are many approaches for conjugating Abs to the QD surface (Table 1), most of them yield site-nonspecific conjugates, where the affinity and specificity of Abs and, hence, the functional efficiency of the conjugates are decreased. At the same time, there are numerous site-specific conjugation methods that have been developed for conjugating Abs with drugs, organic fluorescent dyes, nucleic acids, etc., and can be adapted for site-specific conjugation of Abs with QDs. There is also the problem of a low efficiency of QD–Ab conjugation, whether site-specific or site-nonspecific. Moreover, even in different studies using similar conjugation techniques, the mean number of Abs conjugated to one QD often varies several-fold, or even by orders of magnitude. It should also be noted that there is no versatile approach that would allow highly efficient conjugation of any Ab to the QD surface. On the other hand, sufficiently reproducible results of conjugation are obtained when biotinylated Abs are conjugated to streptavidin-coated QDs and when Abs are conjugated via their amino groups to carboxylated QDs by carbodiimide chemistry methods, or via their thiol groups to QDs coated with ligands containing amino groups. The corresponding conjugation kits are now commercially available. Approaches to site-specific QD–Ab conjugation allow obtaining conjugates with higher affinity for target analytes, but they are still much less developed than the approaches for conjugating Abs with organic fluorescent dyes, nucleic acids, and drugs.

- Methods of QD synthesis, their transfer to the aqueous phase, and functionalization of their surface with organic ligands;
- Methods of preparation and selection of specific Abs for conjugation;
- Comparative data on different adapter molecules and crosslinkers in terms of enhancing QD–Ab conjugation effectiveness;
- Characteristics of the obtained conjugates, including not only the results of testing the conjugate performance in specific techniques but also data on their hydrodynamic diameter, the efficiency of QD–Ab conjugation (number of Abs per QD), changes in the Ab affinity and specificity, as well as the QD optical properties, upon conjugation;
- Data on post-processing of the conjugates for blocking the QD surface and reducing nonspecific binding to biological molecules.

For further development of site-specific conjugation approaches, more studies are required that would provide detailed information on the following issues:

These data would allow a more correct estimation of the dependence of the conjugation efficiency on the use of various adapter molecules and immobilization methods, which would ultimately make it possible to develop a reproducible method for site-specific QD–Ab conjugation and even to market a conjugation kit based on these conjugates. To date, the use of recombinant Abs, which are becoming more available every year, and incorporation of peptide tags or non-canonical amino acid residues into them for conjugation with QDs seems to be one of the most promising approaches to the development of site-specific conjugation methods.

Finally, let us compare different approaches to QD–Ab conjugation from both commercial and technological perspectives. It is evident that site-specific conjugation provides the highest signal level. For instance, Wilkins et al. [90] have demonstrated that site-specific conjugation of Abs via carbohydrates results in an eightfold lower LoD compared to site-nonspecific conjugation using carbodiimide chemistry methods. Zhang et al. [94] have shown that site-specific conjugation of antibody fragments results in a fourfold lower LoD than site-nonspecific conjugation of full-sized antibodies. Additionally, Anderson et al. [97]. have demonstrated that site-specifically conjugated sdAbs produce a four times stronger signal than similar sdAbs conjugated site-nonspecifically using streptavidin–biotin interactions. Thus, site-specific conjugation ensures the highest detection sensitivity, but the ideal conjugation technology should also be inexpensive and easy to scale up. The cost of a conjugation method consists of the cost of directly coupling reagents and the cost of ligand used for QD synthesis and solubilization. Most researchers use commercially available QDs functionalized with carboxyl groups or streptavidin for conjugation with Abs mediated by EDC or streptavidin–biotin. The cost of these QDs is significantly higher than in-house synthesis and solubilization of QDs (we roughly estimate this difference at about an order of magnitude). Furthermore, the use of affinity proteins for site-specific conjugation is more expensive compared to covalent site-nonspecific conjugation because of the additional cost of affinity proteins and additional technological steps. The cost of site-specific antibody conjugation via carbohydrates or disulfide bounds is comparable with that of covalent site-nonspecific conjugation, but the technique is somewhat more complicated. Physical absorption is the least expensive method for conjugation of Abs with nanoparticles, including gold nanoparticles and QDs, because no special chemical reagents are used for conjugation, although charged water-soluble QDs are required. At the same time, His-tag/Ni-NTA interactions are widely used in different laboratory procedures, such as recombinant protein purification, and Ni-NTA is quite inexpensive. In recent years, the number of commercially available recombinant Abs has increased by several orders of magnitude, which has not only reduced their cost, but also made it possible to produce antibodies labeled with a histidine residue (or short chains of histidine residues) with a minimal overhead. This, in turn, makes it possible to perform noncovalent conjugation via metal-affinity interactions between zinc ions of the QD shell and His-tagged Abs, which may be the least expensive and most convenient way to produce QD–Ab conjugates, e.g., for LFAs.

Thus, the combination of modern biotechnologies for obtaining Abs and polymer chemistry for functionalizing the QD surface will ultimately yield reproducible and relatively simple and inexpensive methods for site-specific conjugation of Abs with QDs.

## Figures and Tables

**Table 1:** Examples of methods for site-specific and site-nonspecific conjugations of antibodies to quantum dots.

QD Type	QD Surface Ligand	Conjugation Principle	Comments	Tested Application	Ref.
**Site-specific QD–Ab conjugation**					
CdSe/ZnS; CdSeTe/CdS	GSH	Immunoglobulin-binding domain of protein G (GB1) was expressed with 6 His tags at its N-terminus (HisGB1). HisGB1 directly binds to QDs via complexation between histidine tags and Zn^2+^ or Cd^2+^ ions at the QD surface because of the high affinity of His to these ions.	Since the GB1 domain recognizes the Fc region of IgG, its antigen-binding activity is not affected by Ab-conjugation reactions.	Fluorescent microscopy and in vivo fluorescence imaging	[84]
CdSe/ZnS	DHLA	QDs capped with negatively charged DHLA interact with a mixture of PG-zb and MBP-zb via the positively charged zb peptide tag.	PG interacts with the Fc region of the anti-SEB or anti-TNT Ab. The amount of Abs bound to the QD surface can be controlled by varying the ratio of PG-zb and MBP-zb in the mixture. MBP can be used to purify conjugates.	Fluorescent immunosorbent assay; continuous-flow immunoassay	[85]
CdSe/ZnS	Carboxyl compound	The tripartite fusion protein (His–ELP–PL) consists of an N-terminal His tag used for QD conjugation via strong metal-affinity coordination with Zn^2+^ on the QD surface, the ELP midblock of 78 repeating VPGVG units for stimulus-responsive purification, and the C-terminal PL.	PL has a high affinity for Ig κ-light chains. ELP tag can undergo a reversible phase transition from water-soluble forms into aggregates and can be used for purification of QDs IgGs conjugates.	Western blot analysis; microarrays	[87]
CdSe/ZnS	Amino compound	A photoactivated probe (Bpa) with a Cys handle (an adapter) is integrated into the Z domain of protein A covalently coupled to the Fc region of the Ab using ultraviolet irradiation. This construct is conjugated to QDs via its free Cys sulfhydryl group and amino groups on QD surface in the presence of maleimide.	The method yields site-specific covalent QD–Ab conjugates that have a higher antigen affinity than randomly oriented conjugates.	Immunofluorescence; lateral flow immunoassay	[89]
CdSe/CdZnS	Gluthathione	Protein A coupled to QD surface by a carbodiimide chemistry technique is used for non-covalent conjugation with the Fc fragment of full-length Abs.	The hydrodynamic size of protein A–modified QDs is smaller than 10 nm. These QDs are monodisperse and do not aggregate in PBS over two months.	Multiplexed confocal fluorescence microscopy	[77]
CdSe/ZnS	Streptavidin	Biotin coupled with the divalent ZZ domain based on the B domain of protein A is used as a linker for conjugation of a streptavidin-coated QD and the Fc domain.	The ZZ peptide does not bind the Fab region of Ig, in contrast to all individual domains of protein A, which offers the advantage of a more uniform binding to antibodies (via the Fc region), leaving the Fab domain free to bind its target.	Fluorescent Western blot	[81]
CdS/ZnS	Amino compound	Oxidized glycosylated Fc regions of Abs were used for binding with amino-functionalized QDs, and the resultant conjugate was stabilized with sodium cyanoborohydride.	Site-specific conjugation increased the detection limit by a factor of 8 and enhanced the fluorescent signal compared with conjugation using carbodiimide chemistry.	Lateral flow assay	[90]
CdSe/ZnSe/ZnS	GSH	Succinimidyl valerate–PEG–maleimide (SMPEG) was used for obtaining maleimide-functionalized QDs subsequently conjugated with sulfhydryl groups of Cys in the hinge region of reduced Abs.	The number of Abs conjugated on the surface of one QD by SMPEG-mediated coupling was 2.13 ± 0.5, versus 0.76 ± 0.2 in the case of the site-nonspecific EDC-mediated coupling.	Fluorescent microscopy; dot blot assay	[94]
Qdot^®^ 585 (CdSe/ZnS)	Amino-modified polyethylene glycol	Affinity-purified goat anti-mouse Abs were partially reduced with DTT or 2-MEA and conjugated with SMCC-activated QDs via sulfhydryl groups of the Ab hinge region.	This method allows site-specific conjugation of full-length Abs with QDs without the use of affinity proteins.	Dot blot analysis; flow cytometry	[92]
Qdot^®^ 605 (CdSe/ZnS)	Carboxyl compound	Carboxyl-modified QDs functionalized with maleimide are conjugated, via the reduced Ab –SH groups, with Ab fragments obtained by splitting full-length Abs with tris(2-carboxyetheyl)phosphine hydrochloride.	The QD–Ab conjugates are imaged by high-speed atomic force microscopy. The number of attached Abs varied from 2 to 7 per QD (4.3 ± 1.5 on average).	Fluorescence microscopy	[106]
CdSe/ZnS	PEG derivatives containing terminal hydroxyl	QDs containing hydroxyl group on their surface were conjugated to the sdAbs containing cysteine residue in C-terminus using the PMPI crosslinker	Hydrodynamic diameter of QD-sdAb conjugate was about 12 nm and the contained four molecules of sdAbs on the surface of one QD	Flow cytometry	[91]
CdSe/ZnS	DHLA	sdAb with three repeats of 6 histidine residues separated by short spacer sequences at its C-terminus is expressed and coordinated to zinc ions on the QD surface.	Site-specifically conjugated sdAb–His–QDs provide a 4-fold higher signal in SPR than site-non-specifically conjugated sdAb–biotin–streptavidin–QDs.	Fluorescent immunoassay; SPR	[97]
CdSe/ZnS	SH-, NH_2_-, or OH-modified PEG	sdAb with an additional Cys at its C-terminus is expressed.	Ultrasmall conjugates. The hydrodynamic diameter of QDs functionalized with PEG derivatives is 8.8 nm; that of sdQD–Ab conjugates is 11.9 nm.	Flow cytometry; fluorescence immunohistochemistry	[98]
CdSe/CdS	PAOA	An scFv Ab fragment with the SpyTag peptide at the C-terminus interacts with a SpyCatcher-coated QD.	SpyCatcher proteins spontaneously form covalent isopeptide bonds with SpyTag peptides, thereby forming conjugates of engineered Abs and QDs with controlled stability, orientation, and stoichiometry.	Fluorescence microscopy	[102]
Qdot^®^ 625 (CdSe/ZnS)	Polymer	Qdot^®^ 625 activated with SMCC covalently bind with DTT-reduced scFv fragments.	The scFv–QD conjugates not only serve as detection tags, but also have tumoricidal activity, inhibiting xenograft breast tumor growth in vivo.	Fluorescence immunohistochemistry; Western blot analysis	[104]
Hg_x_Cd_1−x_Se/CdzZn_1−z_S	Polymer P(IM-N_3_)	A full-length Ab is reacted with DBCO–sulfo-N-hydroxysuccinimidyl ester to obtain DBCO bound to the Ab via amide bonds. The Fab′ fragment is conjugated at the hinge region with DBCO–maleimide. The conjugates are formed by covalent click reactions between the azides and cyclooctyne-functionalized Abs.	Conjugates of QDs with full-length Abs have a hydrodynamic diameter of about 22 nm, and conjugates with Fab fragments are only 12.3 nm in size, which is only slightly larger than the size of bare QDs (7.5 nm). It has been shown that smaller bioaffinity agents and site-specific orientation of Abs/Ab fragments on QDs are required to enhance penetration into biospecimens and minimize nonspecific staining.	Fluorescence Immunohistochemistry and microscopy	[105]
**Site-nonspecific QD–Ab conjugation**					
Qdot^®^ 655 (CdSe/ZnS)	Streptavidin directly bound to the polymer shell	Streptavidin on the QD surface binds biotinylated Abs	The size of streptavidin-functionalized QDs is 14.7 ± 3.7 nm; that of QD–Ab conjugates is 29.6 ± 11.8 nm, as estimated by transmission electron microscopy.	Fluorescence quenching upon binding the analyte	[55]
CdTe	3-Mercaptopropionic acid	Sterptavidin is bound to the QD surface by carbodiimide chemistry methods and then conjugated with biotinylated Abs.	The use of QD–Ab conjugates in immunosorbent assay increases the sensitivity of detection of chlorpyrifos 5.5-fold compared to the conventional ELISA using HRP–Ab conjugates.	Fluorescence-linked immunosorbent assay	[57]
Carbon	Carboxyl compound	Amino groups of sdAbs are conjugated with carboxylic group on the QD surface by carbodiimide chemistry methods.	The small allows the sdQD–Ab conjugates diffuse more efficiently into solid tumors. The conjugates are nontoxic for normal human cells and for 4 weeks after injection.	Flow cytometry, immunofluorescent staining	[99]
CdSe/ZnS	DHLA	An avidin-functionalized QD is conjugated with randomly biotinylated Abs.	Functionalization of the QD surface with a mixture of avidin and MBP-leucine zipper interaction domain allows controlling the amount of avidin on the QD surface and, hence, the amount of conjugated Abs per QD.	Sandwich immunoassay	[56]
CdSe/ZnS	Streptavidin	An streptavidin-functionalized QD is conjugated with randomly biotinylated Abs.	The LoD of specific mycobacterial flavoprotein reductase is 12.5 pg/μL.	Fluorescent lateral flow immunoassay	[125]
Qdot^®^ 655 (CdSe/ZnS)	Streptavidin	An streptavidin-functionalized QD is conjugated with randomly biotinylated Abs.	The LoD of cartilage oligomeric matrix protein is 3.125 nM and LoD of human growth hormone—5 nM.	96-well plate and microvolome slide fluorescence-linked immunesorbent assay	[126]
eFluor 490 (CdSe/ZnS)	Carboxyl compound	Carbodiimide-mediated conjugation of full-length Abs with QDs.	A protocol for carbodiimide-mediated QD–Ab conjugation and conjugate optimization is used.	Immunofluorescence imaging; membrane sandwich immunoassay	[62]
CdSe/CdS/ZnS	Polymer coated QDs with carboxyl groups	Carbodiimide-mediated conjugation of full-length Abs.	The LoD of fumonisin B_1_ is 2.8 μg/L.	Fluorescent lateral flow immunoassay	[127]
CdSe/ZnS	Commercial QDs with carboxyl groups	Carbodiimide-mediated conjugation of full-length Abs.	The LoD of ciliary neurotrophic factor is 6.45 pg/mL.	Fluorescent lateral flow immunoassay	[65]
ZnCdSe/ZnS	Commercial QDs with carboxyl groups	Carbodiimide-mediated conjugation of full-length Abs.	The LoD of tiamulin—0.309 pg/mL.	Indirect competitive fluorescence-linked immunosorbent assay	[128]
ZnCdSe/ZnS	Commercial QDs with carboxyl groups	Carbodiimide-mediated conjugation of full-length Abs.	The LoD of apramycin is 0.38 ng/mL.	Fluorescence-linked immunosorbent assay	[63]
QSH_620_	Carboxylic acid-modified QDs with emission at 620 nm	Carbodiimide-mediated conjugation of full-length Abs.	The LoD of Aflatoxin B_1_ is 0.04 ng/mL.	FRET based immunosensor	[67]
CdSe/CdS; CdSe/CdZn_0.3_Zn_0.7_S; InAs/Cd_0.2_Zn_0.8_S	NBPILs	Tetrazine-modified Abs interact with NBPILs-functionalized QDs via tetrazine–norbornene cycloaddition.	The hydrodynamic diameter of the conjugate is 15–17 nm (that of free Abs is 12 nm); the quantum yield of the conjugated QDs is as high as ~80%.	Flow cytometry; in vivo microscopy; multiplexed single-cell imaging in vivo	[70]
CdSe/ZnS	Carboxyl compound	The aldehyde groups of Abs oxidized by sodium periodate are conjugated with the amino groups of BSA-modified QDs.	BSA acts as a QD-stabilizing agent and protect the Ab structure upon conjugation. Addition of BSA reduces nonspecific binding of the conjugates.	Confocal microscopy; fluorometric immunoassay	[72]
CdTe	3-Mercaptopropionic acid	QDs are coated with BSA via the carbonyldiimidazole linker. Anti-CA19-9 Abs are conjugated to BSA via aldehyde groups.	The CA19-9 detection limit is about 1.66 10^−4^ U mL^−1^, which is significantly below the cutoff for early pancreatic cancer risk.	CA19-9 immunosensor based on QD fluorescence quenching	[73]
Qdot^®^ 565 (CdSe/ZnS)	Carboxyl compound	Carbodiimide conjugation of full-length Abs.	Conjugation of QDs with anti-ApoE Abs bound with ApoE-modified magnetic particles protecting the Ab binding sites.	Capillary electrophoresis–laser induced fluorescence	[76]

Abbreviations: QD, quantum dot; Ab, antibody; GSH, glutathione; His, hexahistidine; Ig, immunoglobulin; DHLA, dihydrolipoic acid; PG, IgG-binding β2 domain of streptococcal protein G; zb, basic leucine zipper; MBP, maltose-binding protein; SEB, staphylococcal enterotoxin B; TNT, 2,4,6-trinitrotoluene; ELP, elastin-like polypeptide; PL, protein L; Bpa, p-benzoyl-l-phenylalanine; Cys, cysteine; PBS, phosphate-buffered saline; 2-MEA, 2-mercapto-ethanolamine-HCl; SMCC, N-succinimidyl 4-(maleimidomethyl) cyclohexanecarboxylate; PEG, polyethylene glycol; PMPI, p-maleimidophenyl isocyanate; SPR, surface plasmon resonance; PAOA, poly(acrylic acid)-co-poly(n-octylacrylamide)-co-poly(2-aminoethylacrylamide); DTT, dithiothreitol; DBCO, dibenzylcyclooctyne; ELISA. enzyme-linked immunosorbent assay; HRP, horseradish peroxidase; NBPILs, polyimidazole ligands incorporating norbornene; BSA, bovine serum albumin; CA19-9, carbohydrate antigen 19-9; ApoE, apolipoprotein E; LoD, limit of detection.

## Data Availability

Not applicable.
